# Image-matching digital macro-slide—a novel pathological examination method for microvascular invasion detection in hepatocellular carcinoma

**DOI:** 10.1007/s12072-022-10307-w

**Published:** 2022-03-16

**Authors:** Hong-Ming Yu, Kang Wang, Jin-Kai Feng, Lei Lu, Yu-Chen Qin, Yu-Qiang Cheng, Wei-Xing Guo, Jie Shi, Wen-Ming Cong, Wan Yee Lau, Hui Dong, Shu-Qun Cheng

**Affiliations:** 1 Department of Hepatic Surgery VI, Eastern Hepatobiliary Surgery Hospital, Second Military Medical University, 225 Changhai Road, Shanghai, 200433 China; 2 Clinical Research Center, Changhai Hospital, Second Military Medical University, 168 Changhai Road, Shanghai, 200433 China; 3 Department of Health Statistics, Second Military Medical University, 800 XiangYin Road, Shanghai, 200433 China; 4 Department of Pathology, Eastern Hepatobiliary Surgery Hospital, Second Military Medical University, 225 Changhai Road, Shanghai, 200433 China; 5 Faculty of Medicine, The Chinese University of Hong Kong, Shatin, Hong Kong SAR China

**Keywords:** Hepatocellular carcinoma, Microvascular invasion, 3-Point baseline sampling protocol, 7-Point baseline sampling protocol, Imaging-matching digital macro-slide, Recurrence-free survival, Receiver operating characteristic curve, AFP, PIVKA-II

## Abstract

**Background:**

Microvascular invasion (MVI) is a prominent risk factor of postoperative recurrence for hepatocellular carcinoma (HCC). The MVI detection rate of conventional pathological examination approaches is relatively low and unsatisfactory.

**Methods:**

By integrating pathological macro-slide with whole-mount slide imaging, we first created a novel pathological examination method called image-matching digital macro-slide (IDS). Surgical samples from eligible patients were collected to make IDS. The MVI detection rates, tumor recurrence rates and recurrence-free survival were compared among conventional 3-Point and 7-Point baseline sampling protocols and IDS. Additionally, biomarkers to recognize MVI false negative patients were probed via combining conventional pathological sampling protocols and IDS. Receiver operating characteristic curve (ROC) analysis was used to obtain the optimal cutoff of biomarkers to distinguish MVI false negative patients.

**Results:**

The MVI detection rates were 21.98%, 32.97% and 63.74%, respectively, in 3-Point, 7-Point baseline sampling protocols and IDS (*p* < 0.001). Tumor recurrence rate of patients with MVI negative status in IDS (6.06%) was relatively lower than that of patients with MVI negative status in 3-Point (16.90%) and 7-Point (16.39%) sampling protocols. Alpha-fetoprotein (AFP) and protein induced by vitamin K absence or antagonist-II (PIVKA-II) were selected as potential biomarkers to distinguish MVI false negative patients.

**Conclusions:**

Our study demonstrated that IDS can help enhance the detection rate of MVI in HCC and refine the prediction of HCC prognosis. Alpha-fetoprotein is identified as a suitable and robust biomarker to recognize MVI false-negative patients in conventional pathological protocols.

**Supplementary Information:**

The online version contains supplementary material available at 10.1007/s12072-022-10307-w.

## Introduction

Hepatocellular carcinoma (HCC) is the fourth leading cause of cancer-related mortality worldwide and the leading cause of death among patients with cirrhosis [1]. HCC is characterized by an aggressive clinical course and dismal outcomes, with about two-thirds of patients diagnosed at advanced stage at first presentation. Up to 70–80% of HCC patients will develop disease recurrence within five years following initial curative treatments [1].

Microvascular invasion (MVI) refers to the presence of tumor cell clusters in a vascular lumen lined by endothelial cells under microscopic examination [2, 3]. It has been repeatedly demonstrated to be a prominent risk factor of postoperative recurrence for HCC patient [4, 5]. It is well known that pathological examination is the gold standard to diagnose and grade cancers via observing the morphological character, differentiation degree and growth pattern of tumor cells. Besides, pathological examination can detect some important biological factors around tumor tissues, including micro-satellites and MVI. Nevertheless, due to the limited scope of routine glass slides and randomness and bias of sampling, conventional pathological testing protocol has a tendency to under-report the incidence of MVI in HCC. Hence, constructing a novel pathological technique with greater MVI detection power is required.

Recently, based upon development of histological slide digitization and computational image processing, whole-mount slides imaging (WSI) has been popularized in oncology studies. WSI refers to scanning a complete microscope slide, capturing many small high-resolution image tiles or strips and then montaging them to create a full image of a histopathological section. Many studies have highlighted the clinical significance of WSI in assisting pathologists investigating the whole spectrum of tumor biopsy specimens, and in identifying prognostic markers and histological subtypes of various cancers [6–8]. Macroscopic histological slide (macro-slide), different from routine small slides, expands to cover the whole section of tumor tissues and maintains the integrity of tumor specimens to the greatest extent. Macro-slide has the advantage to exhibit more pathological features compared with conventional small slides.

In this study, by combining macro-slide and WSI technique, we create and first report the clinical utility of a novel pathological examination method called “image-matching digital macro-slide (IDS)” for MVI detection in HCC. We show that IDS has the capacity to remarkably increase MVI detection rates in HCC, guiding postoperative adjuvant therapies and surveillance protocols, thus reducing long-term recurrence rates. By analyzing IDS, we are able to get deeper insight into the comprehensive and most relevant features of tumors.

## Methods and materials

### Patients

Consecutive patients who underwent radical hepatectomy of liver cancer at Eastern Hepatobiliary Surgery Hospital (EHBH) from October 2018 to December 2019 were enrolled. The follow-up date was censored on January 31, 2021. The inclusion criteria were as follows: (I) patients aged from 20 to 70 years old, (II) Child–Pugh class A–B7, (III) Eastern Cooperative Oncology Group (ECOG) performance score was 0–1, and (IV) underwent radical resection and had complete postoperative histopathological tissues. The exclusion criteria were as follows: (I) palliative-intend resection, (II) presence of extrahepatic metastasis or major vascular invasion, (III) underwent preoperative anti-cancer treatment, (IV) a history of other malignant tumors, and (V) pathologically confirmed intrahepatic cholangiocarcinoma (ICC) or combined HCC–ICC. The study was approved by institutional review board of our hospital. Written informed consent was obtained from patients for their data to be used for research purposes.

### Pathological diagnosis standard

Two experienced pathologists identified HCC and MVI in all cases, and a third pathologist participated in the identification and gave the final result when there was ambiguity. HCC was diagnosed upon the Guidelines for the Diagnosis and Treatment of Hepatocellular Carcinoma (2019 Edition) [9]. MVI was defined as a cancer cell cluster composed of ≥ 50 cells in a microscopic vessel adjacent to the primary tumor [10, 11].

### Materials

The following materials were required in this study: (I) pathological sampling console, paraffin embedding station (Leica Biosystems Richmond, Inc., USA), paraffin slicing machine, cooler, dehydrator, slice drying machine, and high-resolution slice scanner; (II) Tissue embedding box (7.7 cm × 4.3 cm × 1.3 cm), custom-made anti-off slide glass (7.5 cm × 5.0 cm), and custom-made cover glass (6.0 cm × 5.0 cm); (III) special paraffin embedding mold (Leica Biosystems Richmond, Inc., USA), large paraffin block holder, and special cutter head; (IV) Olympus Automatic Digital Pathology Scanner (VS120). The customized items or equipment were shown in Fig. S1.

### Intraoperative marking

When intraoperative exploration, we determined the surgical resection range and marked the cutting edge and direction. It was recommended that the cross-section of the human body be taken as the sampling plane, marked with two asymmetric directions, and photographed for filing. Consequently, it was more convenient to distinguish the direction of the tumor specimen in vitro.

### Specimen processing

**Fig. 1:**
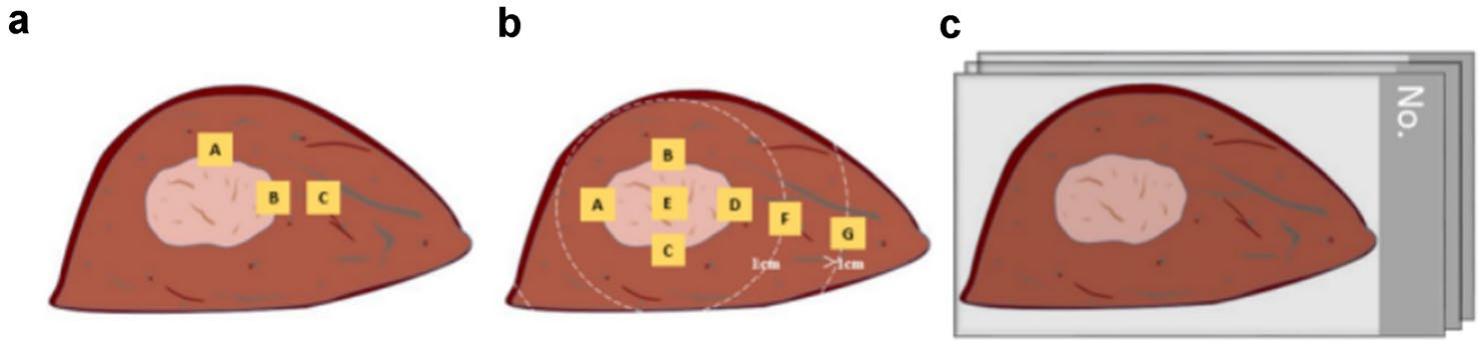
The model of different pathological sampling methods: **a** 3-Point baseline sampling protocol. **b** 7-Point baseline sampling protocol. **c** IDS sampling protocol

We determined the section according to the maximum diameter of the tumor, half of which were taken according to the 3-Point and 7-Point baseline sampling protocols (Fig. 1a; Fig. 1b), the other half of which were taken using the IDS method (Fig. 1c). After the specimen was detached, 10% neutral formaldehyde should be injected as soon as possible (within 30 min) and fixed continuously for not less than 48 h. According to the direction of sampling and section, the thickness of the specimen was 0.5–1.0 cm, and the maximum size of the trimmed specimen was 6.0 cm × 5.0 cm. If the specimen was too large, it could be divided into several parts as required.

### Grossing and paraffin embedding

The specimens were rinsed for formaldehyde with water for at least 2 h. Tissue automatic dehydrator was used to dehydrate specimens, which were then dried using slice drying machine. Next, the specimens were put into and soaked the liquid paraffin. The tissue specimens were embedded and shaped using paraffin embedding station and special paraffin embedding mold. The required sections were confirmed and adjusted again when shaping the tissues.

### Serial sectioning and Hematoxylin–Eosin (HE) staining

When serial sectioning, the paraffin block was fixed with large holder. Paraffin slicing machine and matched special cutter head for big paraffin block were used to make sections with a thickness of 3 μm. After fished out, the slides were baked at 75 °C for 15 min with a baking machine, so that the tissues and the slides can be firmly adhered. The sections were stained with hematoxylin and eosin for histological examination.

### Digital scanning and analysis

All tissue sections were scanned with a high-resolution slice scanner and corresponding image data were stored. The images were analyzed using the software matched with the scanner for parameters such as micro-metastases, stromal cell proportion, peripheral inflammatory changes, etc. The image data were matched with the scanned images, and the characteristics of pathological and imaging changes were analyzed again. Digital image signals in the region could be analyzed according to the research content.

### Follow-up

Patients were followed up with laboratory tests including tumor biomarkers and liver biochemistry, abdominal ultrasonography, and contrast-enhanced CT once every 3–6 months. The diagnosis of intrahepatic recurrence was made by imaging findings alone if the tumor displayed typical enhancement characteristics; otherwise, the recurrent diseases were biopsied. Recurrence-free survival (RFS) time was calculated from the date of first surgery to the date when there was a clear evidence of recurrent disease.

### Statistical analysis

Continuous variables were expressed as mean (standard deviation) or median (interquartile range) and compared using student’s *t* test or Mann–Whitney *U* test according to the distribution of variables. Categorical variables were compared using chi-square test or Fisher’s exact test. Survival curves were generated using the Kaplan–Meier method and compared using the log-rank test. Receiver operating characteristic curve (ROC) analysis was used to obtain the optimal cutoff value of AFP and PIVKA-II to distinguish MVI false negative patients. Clinical performance of 3-Piont, 7-Point and IDS to identify MVI actual positive was assessed by the sensitivity, specificity, and predictive values. Statistical analyses were performed by SPSS 26.0 software (SPSS, Inc., Chicago, IL, USA) and R 3.6.3 software (R Development Core Team). p values less than 0.05 indicated statistical significance.

## Results

### The detailed process of IDS

**Fig. 2:**
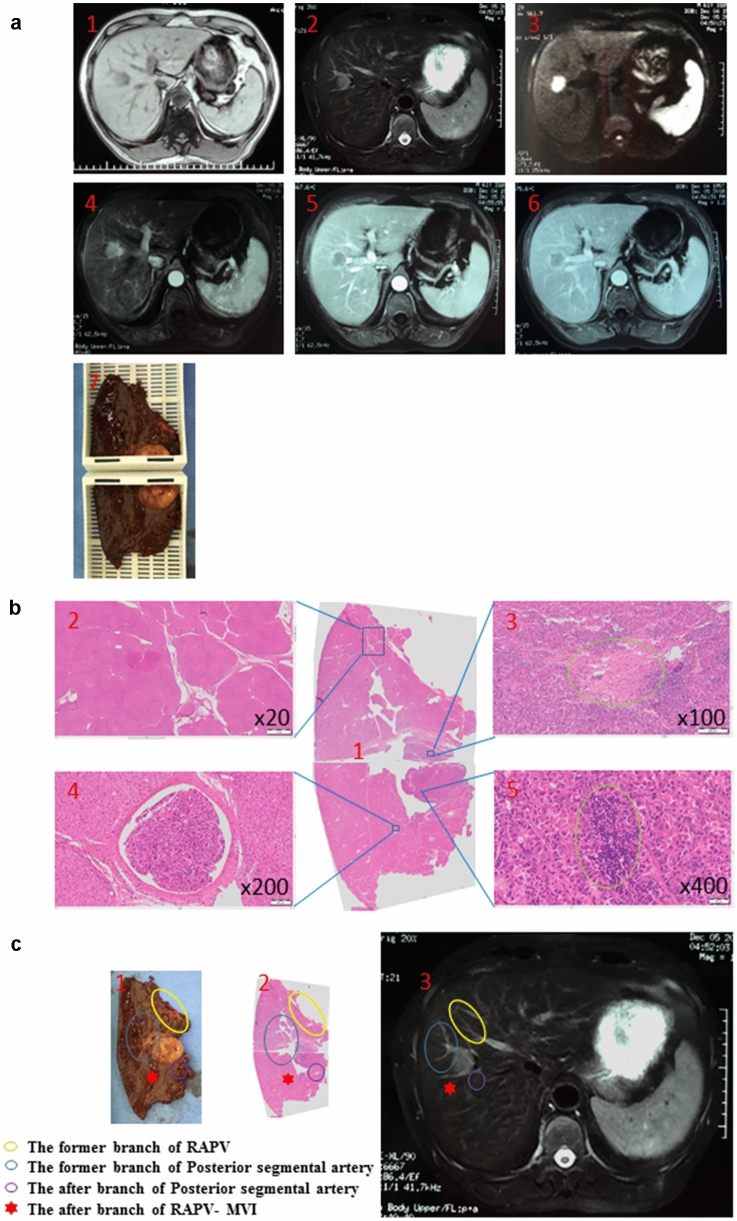
A case of image-matching digital macro-slide. **a** Preoperative MRI and sample selection of the patient: 1, T1WI; 2, T2WI; 3, DWI; 4, arterial phase; 5, portal phase; 6, delayed phase. **b** image after WSI scanning: 1, making macro-slide samples; 2, the overall view of the image after the sample is scanned and stitched; 3, mixed nodular cirrhosis was observed after magnification of 20 times; 4, local hemorrhagic necrosis was seen inside the tumor after magnification of 100 times; 5, MVI was observed after magnification of 200 times; 6, accumulation of inflammatory cells in the tumor was observed after 400-fold magnification. **c** corresponding specimen and digital macro-slide combined to MRI imaging (T2WI): 1, specimen; 2, digital macro-slide; 3, MRI imaging (T2WI), in 1: 1: 1. Marking the different vessels and MVI locations with different colors in the panel

Preoperative MRI showed that the tumor was located in the posterior lower segment of the right lobe of the liver. The size of the tumor was 72 mm × 58 mm × 55 mm. T2W1 showed slightly higher, equal, and uneven equal-high signals. Scanning after enhancement, the hepatic artery phase demonstrated obvious uneven enhancement. In the portal phase and delayed phase, the relative signal attenuation was in line with the primary liver cancer (Fig. 2a). The preoperative evaluation was in accordance with the inclusion criteria. During the operation, the tumor was completely resected (R0 resection), and the whole specimen was taken to produce macro-slide. After hematoxylin–eosin staining, the macro-slide was observed at different positions with various magnifications (Fig. 2b). Then, the pathological macro-slide was matched with tumor specimen and imaging data to obtain WSI including MVI positions (Fig. 2c). This total process was named as IDS. The detailed preparation and production process of IDS is shown in the flow diagram (Fig. S2). Another two representative HCC cases with histopathological details focused on MVI are shown in Fig. S3.

### The baseline characteristics and long-term outcomes of eligible patients

A total of 110 primary liver cancer patients were collected in this study. 19 patients who were pathologically diagnosed as intrahepatic cholangiocarcinoma were excluded, and 91 HCC patients were finally included. Using surgically resected specimens from 91 HCC patients, 145 thick slices were produced and examined. Conventional pathology covers tumor in all three extents. In this study, 300 sections in 3-Point, 664 sections in 7-Point, and 145 sections in IDS were examined and compared.

The baseline clinicopathological characteristics are shown in Table 1. Almost all patients had hepatitis B or C virus infection background. The percentage of hepatitis B virus infection was 96.7%, and only one patient did not have hepatitis. The liver function of patients was all graded as Child–Pugh class A. The median tumor size was 3.80 cm. In 4 HCC patients associated with portal vein tumor thrombus (PVTT), PVTT existed in the branches of main portal vein and could be resected radically. The shortest and longest follow-up times were 13 and 28 months, respectively. 24 (26.37%) patients developed disease recurrence and 5 patients (5.49%) died during follow-up.

**Table 1:** Baseline characteristics of patients with hepatocellular carcinoma

Variables	Total (*n* = 91)
Gender, *n* (%)	
Female	13 (14.29)
Male	78 (85.71)
Age (year), Mean ± SD	56.58 ± 9.30
HBsAg, *n* (%)	
Negative	14 (15.38)
Positive	77 (84.62)
HBeAg, *n* (%)	
Negative	74 (81.32)
Positive	17 (18.68)
HBcAb, *n* (%)	
Negative	3 (3.30)
Positive	88 (96.70)
HBV-DNA, *n* (%)	
< 1000 copies/mL	53 (58.24)
≥ 1000 copies/mL	38 (41.76)
HCV, *n* (%)	
Negative	87 (95.60)
Positive	4 (4.40)
AFP, Median (Q1, Q3), ug/L	26.70 (5.45, 511.25)
PIVKA-II, Median (Q1, Q3), mAU/mL	274.00 (62.50, 1172.00)
CA199, Median (Q1, Q3), U/mL	15.50 (8.65, 28.60)
CEA, Median (Q1, Q3), ug/L	2.60 (1.70, 3.40)
ALT, Median (Q1, Q3), U/L	25.00 (16.00, 44.00)
AST, Median (Q1, Q3), U/L	25.00 (18.00, 38.00)
Total bilirubin, Median (Q1, Q3), umol/L	14.30 (10.85, 18.10)
GGT, Median (Q1, Q3), U/L	39.00 (25.00, 77.00)
Albumin, Mean ± SD, g/L	42.90 ± 4.45
GLU, Median (Q1, Q3), mmol/L	5.12 (4.79, 5.65)
ALP, Median (Q1, Q3), U/L	77.00 (59.50, 98.00)
WBC, Median (Q1, Q3), 10^9^/L	4.81 (3.73, 5.72)
RBC, Mean ± SD, 10^12^/L	4.57 ± 0.47
HGB, Mean ± SD, g/L	140.80 ± 15.47
PLT, Mean ± SD, 10^9^/L	149.08 ± 61.11
PT, Median (Q1, Q3), s	11.50 (11.00, 12.05)
Tumor size, Median (Q1, Q3), cm	3.80 (2.50, 5.50)
Tumor number, *n* (%)	
1	83 (91.21)
2	7 (7.69)
4	1 (1.10)
PVTT, *n* (%)	
Absence	87 (95.60)
Presence	4 (4.40)
Encapsulation, *n* (%)	
No	23 (25.27)
Incomplete	42 (46.15)
Complete	26 (28.57)
Liver cirrhosis, *n* (%)	
No	38 (41.76)
Yes	53 (58.24)
Child–Pugh class A, *n* (%)	91 (100.00)
Recurrence, *n* (%)	
No	67 (73.62)
Yes	24 (26.37)

*HBsAg* hepatitis B surface antigen, *HBeAg* hepatitis B e antigen, *HBcAb* hepatitis B core antibody, *HBV-DNA* hepatitis B virus-deoxyribonucleic acid, *HCV* hepatitis C virus, *AFP* alpha-fetoprotein, *PIVKA-II* protein induced by vitamin K antagonist-II, *CA199* carbohydrate antigen199, *CEA* carcinoembryonic antigen, *ALT* alanine aminotransferase, *AST* aspartate aminotransferase, *GGT* γ-glutamyltransferase, *GLU* glucose, *ALP* alkaline phosphatase, *WBC* white blood cells, *RBC* red blood cells, *PLT* platelet, *PT* prothrombin time, *PVTT* portal vein tumor thrombus

### MVI detection rates in 3-Point, 7-Point baseline sampling protocols and IDS

**Fig. 3:**
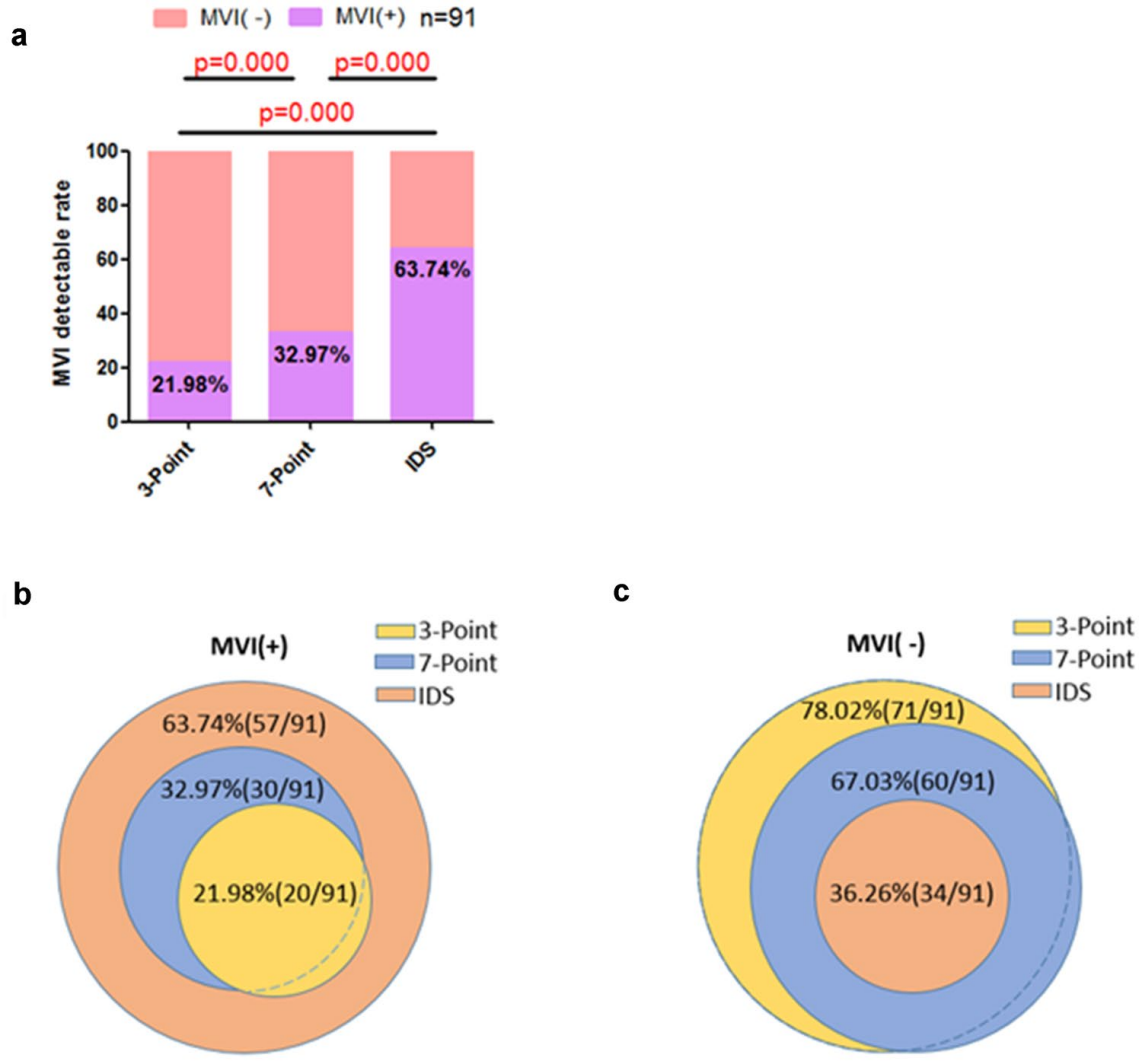
**a** MVI detection rate under 3-point baseline sampling protocol, 7-point baseline sampling protocol and image matching digital macro-slide. **b**, **c** the composition of MVI positive and negative status patients in different detective methods (3-point baseline sampling protocol, 7-point baseline sampling protocol and image matching digital macro-slide)

As shown in Fig. 3a, the detection rates of MVI were 21.98%, 32.97% and 63.74%, respectively, in 3-Point, 7-Point and IDS (*p* < 0.001). Patients with MVI positive status in 3-Point and 7-Point were all included in MVI positive status in IDS. The populations of 3-Point and 7-Point were not exactly the same (Fig. 3b). Among patients with MVI negative status in 3-Point and 7-Point, the two populations were partly different, but they all included MVI negative status in IDS (Fig. 3c). Therefore, in this study, the specificity and sensitivity of IDS on MVI detection were both 100%, while the specificity of 3-Point and 7-Point on MVI detection were both 100%, and the sensitivity of 3-Point and 7-Point on MVI detection were only 34% and 52%, respectively (Table 2). The above results showed that IDS had superior sensitivity and specificity for the detection of MVI than 3-Point and 7-Point.

**Table 2:** MVI sensitivity and specificity under 3-Piont, 7-Point and IDS

Metrics	3-Point	7-Point
Accuracy	0.58 (0.47–0.68)	0.69 (0.59–0.78)
Sensitivity	0.34 (0.22–0.48)	0.52 (0.38–0.65)
Specificity	1.00 (0.89–1.00)	1.00 (0.89–1.00)
Positive predictive value	1.00 (0.83–1.00)	1.00 (0.88–1.00)
Negative predictive value	0.46 (0.35–0.59)	0.54 (0.41–0.67)

*MVI* microvascular invasion, *3-Point* 3-point baseline sampling protocol, *7-Point* 7-point baseline sampling protocol, *IDS* image-matching digital macro-slide

### Tumor recurrence rates in three various pathological examination methods

**Fig. 4:**
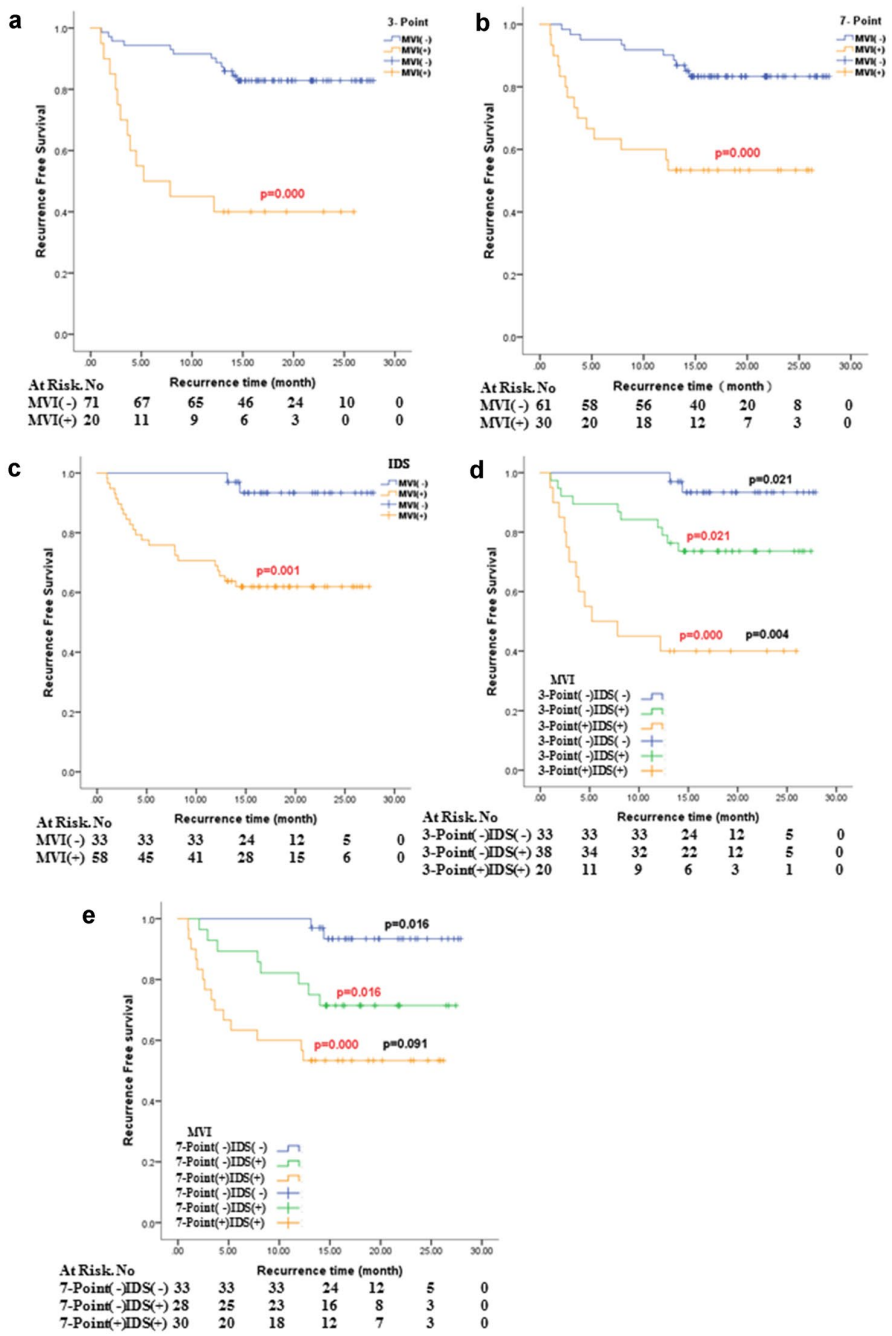
MVI related recurrence-free survival. **a** MVI detected by 3-point baseline sampling protocol and related recurrence-free survival. **b** MVI detected by 7-point baseline sampling protocol and related recurrence-free survival. **c** MVI detected by image matching digital macro-slide and related recurrence-free survival. **d** 3-point baseline sampling protocol combined with image matching digital macro-slide to detect MVI and related recurrence-free survival. **e** 7-point baseline sampling protocol combined with image matching digital macro-slide to detect MVI and related recurrence-free survival

To compare the impact of MVI, which was detected by 3-Point, 7-Point and IDS, respectively, on tumor recurrence, survival analyses were performed (Fig. 4a–c). Under the three methods, patients with MVI positive status were more likely to relapse (*p* < 0.001, *p* < 0.001, *p* = 0.001, respectively). We found that 12 (16.90%) HCC patients with MVI negative status in 3-Point had tumor recurrence, and 2 patients died due to disease progression. In 7-Point sampling method, 10 (16.39%) patients with MVI negative status had recurrence (relapse time range: 2.1–14.4 months), including 2 recurrence-related deaths. In IDS, 2 (6.06%) patients with MVI negative status recurred (relapse time: 13.13 and 14.40 months), and no deaths occurred. Next, we did subgroup analysis combining IDS with 3-Point and 7-Point. Results showed that patients with MVI positive status in both 3-Point and IDS, and in both 7-Point and IDS were most prone to recur. Patients with MVI positive status in IDS were more likely to relapse than patients with actual MVI negative status (*p* = 0.021, *p* = 0.016). (Fig. 4d–e). It is to say, 3-Point and 7-Point sampling protocols have a potential possibility to miss MVI, and patients with MVI false negative status in 3-Point and 7-Point are more likely to recur than patients with actual MVI negative status in all three MVI pathological testing methods.

### Identification of potential biomarkers to distinguish MVI false negative patients in conventional pathological sampling protocols

In order to find out the clinicopathological characteristics of patients with missed MVI in 3-Point and 7-Point, IDS was matched with 3-Point and 7-Point. As shown in Table 3, there were significant differences in 3-Point in AFP, PIVKA-II, ALP, and tumor number. The medians of AFP between 3-Point negative IDS negative and 3-Point negative IDS positive groups were 6.10 (3.10, 20.30) ug/L and 81.30 (10.12, 560.65) ug/L. The medians of PIVKAII between 3-Point negative IDS negative and 3-Point negative IDS positive groups were 107.00 (33.00, 412.00) mAU/mL and 449.00 (90.50, 3168.75) mAU/mL. The medians of ALP between 3-Point negative IDS negative and 3-Point negative IDS positive groups were 79.00 (63.00, 92.00) U/L and 64.50 (54.25, 79.25) U/L. As shown in Table 4, only AFP and PIVKA-II had significant differences in 7-Point. The medians of AFP between 7-Point negative IDS negative and 7-Point negative IDS positive groups were 6.10 (3.10, 20.30) ug/L and 160.55 (20.92, 1210.00) ug/L. The medians of PIVKA-II between 7-Point negative IDS negative and 7-Point negative IDS positive groups were 107.00 (33.00, 412.00) mAU/mL and 460.50 (239.25, 3084.25) mAU/mL, respectively. It revealed that AFP and PIVKA-II could be potential biomarkers to distinguish MVI false negative patients in 3-Point and 7-Point.

**Table 3:** The clinicopathological features were compared in different combinations of 3-Point and IDS with MVI or not

Variables	3-Point (−) IDS (−) (*n* = 33)	3-Point (−) IDS (+) (*n* = 38)	3-Point (+) IDS (+) (*n* = 20)	*p*
Gender, *n* (%)				0.690
Female	4 (30.77)	7 (53.85)	2 (15.38)	
Male	29 (37.18)	31 (39.74)	18 (23.08)	
Age (year), Mean ± SD	57.09 ± 8.96	56.84 ± 9.50	55.25 ± 9.80	0.768
HBsAg, *n* (%)				0.603
Negative	7 (50.00)	5 (35.71)	2 (14.29)	
Positive	26 (33.77)	33 (42.86)	18 (23.38)	
HBeAg, *n* (%)				0.654
Negative	28 (37.84)	29 (39.19)	17 (22.97)	
Positive	5 (29.41)	9 (52.94)	3 (17.65)	
HBcAb, *n* (%)				0.603
Negative	2 (66.67)	1 (33.33)	0 ( 0.00)	
Positive	31 (35.23)	37 (42.05)	20 (22.73)	
HBV-DNA, *n* (%)				0.921
< 1000 copies/mL	20 (37.74)	22 (41.51)	11 (20.75)	
≥ 1000 copies/mL	13 (34.21)	16 (42.11)	9 (23.68)	
HCV, *n* (%)				0.826
Negative	31 (35.63)	37 (42.53)	19 (21.84)	
Positive	2 (50.00)	1 (25.00)	1 (25.00)	
AFP, Median (Q1, Q3), ug/L	6.10 (3.10, 20.30)	81.30 (10.12, 560.65)	346.35 (31.43, 1210.00)	< 0.001
PIVKA- II, Median (Q1, Q3), mAU/mL	107.00 (33.00, 412.00)	449.00 (90.50, 3168.75)	290.50 (137.50, 866.25)	0.032
CA199, Median (Q1, Q3), U/mL	14.00 (6.60, 21.10)	17.50 (9.50, 28.52)	18.00 (9.35, 36.15)	0.263
CEA, Median (Q1, Q3), ug/L	2.80 (2.00, 3.70)	2.70 (1.90, 3.27)	1.70 (1.37, 3.23)	0.168
ALT, Median (Q1, Q3), U/L	25.00 (15.00, 50.00)	20.50 (14.25, 33.75)	27.50 (21.50, 54.25)	0.199
AST, Median (Q1, Q3), U/L	25.00 (17.00, 36.00)	21.50 (18.00, 37.50)	32.50 (23.00, 39.00)	0.222
Total bilirubin, Median (Q1, Q3), umol/L	15.30 (12.20, 18.10)	14.10 (11.57, 17.53)	14.05 (9.50, 20.38)	0.788
GGT, Median (Q1, Q3), U/L	38.00 (25.00, 71.00)	32.00 (24.00, 61.75)	52.00 (39.25, 103.00)	0.069
Albumin, Mean ± SD, g/L	43.09 ± 3.74	42.32 ± 4.65	43.71 ± 5.15	0.505
GLU, Median (Q1, Q3), mmol/L	5.24 (4.89, 6.07)	5.03 (4.67, 5.44)	5.29 (5.01, 5.95)	0.231
ALP, Median (Q1, Q3), U/L	79.00 (63.00, 92.00)	64.50 (54.25, 79.25)	89.50 (72.25, 103.50)	0.017
WBC, Median (Q1, Q3), 10^9^/L	4.87 (3.97, 6.26)	4.81 (3.73, 5.64)	4.54 (3.29, 5.74)	0.587
RBC, Mean ± SD, 10^12^/L	4.53 ± 0.48	4.60 ± 0.42	4.60 ± 0.57	0.779
HGB, Mean ± SD, g/L	141.33 ± 14.19	140.58 ± 16.48	140.35 ± 16.25	0.969
PLT, Mean ± SD, 10^9^/L	159.39 ± 71.71	149.55 ± 54.17	131.15 ± 52.85	0.267
PT, Median (Q1, Q3), s	11.50 (10.90, 11.90)	11.50 (11.00, 12.12)	11.60 (11.07, 12.15)	0.697
Tumor size, Median (Q1, Q3), cm	3.50 (2.50, 4.30)	3.65 (2.08, 6.12)	5.00 (3.50, 6.08)	0.127
Tumor number, *n* (%)				0.009
1	33 (39.76)	35 (42.17)	15 ( 18.07)	
2	0 ( 0.00)	3 (42.86)	4 ( 57.14)	
4	0 ( 0.00)	0 ( 0.00)	1 (100.00)	
PVTT, *n* (%)				0.203
Absence	33 (37.93)	36 (41.38)	18 (20.69)	
Presence	0 ( 0.00)	2 (50.00)	2 (50.00)	
Encapsulation, *n* (%)				0.195
No	4 (17.39)	13 (56.52)	6 (26.09)	
Incomplete	16 (38.10)	16 (38.10)	10 (23.81)	
Complete	13 (50.00)	9 (34.62)	4 (15.38)	
Liver cirrhosis, *n* (%)				0.624
No	13 (34.21)	18 (47.37)	7 (18.42)	
Yes	20 (37.74)	20 (37.74)	13 (24.53)	
Child–Pugh class A, *n* (%)	33 (36.26)	38 (41.76)	20 (21.98)	1.000

*3-Point* 3-point baseline sampling protocol, *MVI* microvascular invasion, *IDS* image-matching digital macro-slide, *HBsAg* hepatitis B surface antigen, *HBeAg* hepatitis B e antigen, *HBcAb* hepatitis B core antibody, *HBV-DNA* hepatitis B virus-deoxyribonucleic acid, *HCV* hepatitis C virus, *AFP* alpha-fetoprotein, *PIVKA-II* protein induced by vitamin K antagonist-II, *CA199* carbohydrate antigen199, *CEA* carcinoembryonic antigen, *ALT* alanine aminotransferase, *AST* aspartate aminotransferase, *GGT* γ-glutamyltransferase, *GLU* glucose, *ALP* alkaline phosphatase, *WBC* white blood cells, *RBC* red blood cells, *PLT* platelet, *PT* prothrombin time, *PVTT* portal vein tumor thrombus

**Table 4:** The clinicopathological features were compared in different combinations of 7-Point and IDS with MVI or not

Variables	7P (−) MS (−) (*n* = 33)	7P (−) MS (+) (*n* = 28)	7P (+) MS ( +) (*n* = 30)	*p*
Gender, *n* (%)				0.147
Female	4 (30.77)	7 (53.85)	2 (15.38)	
Male	29 (37.18)	21 (26.92)	28 (35.90)	
Age (year), Mean ± SD	57.09 ± 8.96	57.32 ± 10.30	55.33 ± 8.86	0.670
HBsAg, *n* (%)				0.474
Negative	7 (50.00)	4 (28.57)	3 (21.43)	
Positive	26 (33.77)	24 (31.17)	27 (35.06)	
HBeAg, *n* (%)				0.267
Negative	28 (37.84)	20 (27.03)	26 (35.14)	
Positive	5 (29.41)	8 (47.06)	4 (23.53)	
HBcAb, *n* (%)				0.641
Negative	2 (66.67)	1 (33.33)	0 (0.00)	
Positive	31 (35.23)	27 (30.68)	30 (34.09)	
HBV-DNA, *n* (%)				0.942
< 1000 copies/mL	20 (37.74)	16 (30.19)	17 (32.08)	
≥ 1000 copies/mL	13 (34.21)	12 (31.58)	13 (34.21)	
HCV, *n* (%)				0.458
Negative	31 (35.63)	26 (29.89)	30 (34.48)	
Positive	2 (50.00)	2 (50.00)	0 (0.00)	
AFP, Median (Q1, Q3), ug/L	6.10 (3.10, 20.30)	160.55 (20.92, 1210.00)	66.35 (13.67, 1210.00)	< 0.001
PIVKA-II, Median (Q1, Q3), mAU/mL	107.00 (33.00, 412.00)	460.50 (239.25, 3084.25)	257.00 (90.50, 725.75)	0.021
CA199, Median (Q1, Q3), U/mL	14.00 (6.60, 21.10)	19.50 (11.17, 30.35)	15.40 (8.62, 33.60)	0.336
CEA, Median (Q1, Q3), ug/L	2.80 (2.00, 3.70)	2.70 (2.08, 3.12)	1.90 (1.33, 3.53)	0.170
ALT, Median (Q1, Q3), U/L	25.00 (15.00, 50.00)	22.50 (16.25, 36.50)	25.00 (17.25, 46.75)	0.853
AST, Median (Q1, Q3), U/L	25.00 (17.00, 36.00)	24.00 (18.00, 39.50)	26.50 (18.25, 37.75)	0.936
Total bilirubin, Median (Q1, Q3), umol/L	15.30 (12.20, 18.10)	13.55 (11.38, 17.88)	15.05 (9.85, 19.00)	0.789
GGT, Median (Q1, Q3), U/L	38.00 (25.00, 71.00)	32.00 (24.00, 63.00)	45.00 (30.50, 86.00)	0.471
Albumin, Mean ± SD, g/L	43.09 ± 3.74	41.79 ± 4.70	43.74 ± 4.84	0.242
GLU, Median (Q1, Q3), mmol/L	5.24 (4.89, 6.07)	5.21 (4.94, 5.68)	5.02 (4.75, 5.40)	0.392
ALP, Median (Q1, Q3), U/L	79.00 (63.00, 92.00)	66.50 (54.75, 83.00)	79.00 (60.75, 101.75)	0.246
WBC, Median (Q1, Q3), 10^9^/L	4.87 (3.97, 6.26)	4.43 (3.43, 5.54)	5.02 (3.66, 5.64)	0.473
RBC, Mean ± SD, 10^12^/L	4.53 ± 0.48	4.56 ± 0.41	4.64 ± 0.52	0.611
HGB, Mean ± SD, g/L	141.33 ± 14.19	139.46 ± 15.06	141.47 ± 17.50	0.862
PLT, Mean ± SD, 10^9^/L	159.39 ± 71.71	144.00 ± 57.54	142.47 ± 51.41	0.481
PT, Median (Q1, Q3), s	11.50 (10.90, 11.90)	11.55 (11.15, 12.70)	11.45 (11.00, 11.88)	0.610
Tumor size, Median (Q1, Q3), cm	3.50 (2.50, 4.30)	4.00 (2.45, 6.32)	4.65 (2.73, 6.00)	0.254
Tumor number, *n* (%)				0.053
1	33 (39.76)	24 (28.92)	26 (31.33)	
2	0 (0.00)	4 (57.14)	3 (42.86)	
4	0 (0.00)	0 (0.00)	1 (100.00)	
PVTT, *n* (%)				0.153
Absence	33 (37.93)	27 (31.03)	27 (31.03)	
Presence	0 (0.00)	1 (25.00)	3 (75.00)	
Encapsulation, *n* (%)				0.181
No	4 (17.39)	8 (34.78)	11 (47.83)	
Incomplete	16 (38.10)	13 (30.95)	13 (30.95)	
Complete	13 (50.00)	7 (26.92)	6 (23.08)	
Liver cirrhosis, *n* (%)				0.942
No	13 (34.21)	12 (31.58)	13 (34.21)	
Yes	20 (37.74)	16 (30.19)	17 (32.08)	
Child–Pugh class A, *n* (%)	33 (36.26)	28 (30.77)	30 (32.97)	1.000

*7-Point* 7-point baseline sampling protocol, *MVI* microvascular invasion, *IDS* image matching digital macro-slide, *HBsAg* hepatitis B surface antigen, *HBeAg* hepatitis B e antigen, *HBcAb* hepatitis B core antibody, *HBV-DNA* hepatitis B virus-deoxyribonucleic acid, *HCV* hepatitis C virus, *AFP* alpha-fetoprotein, *PIVKA-II* protein induced by vitamin K antagonist-II, *CA199* carbohydrate antigen199, *CEA* carcinoembryonic antigen, *ALT* alanine aminotransferase, *AST* aspartate aminotransferase, *GGT* γ-glutamyltransferase, *GLU* glucose, *ALP* alkaline phosphatase, *WBC* white blood cells, *RBC* red blood cells, *PLT* platelet, *PT* prothrombin time, *PVTT* portal vein tumor thrombus

### Comparison of sensitivity and specificity of AFP and PIVKA-II in identifying MVI false negative patients

**Fig. 5:**
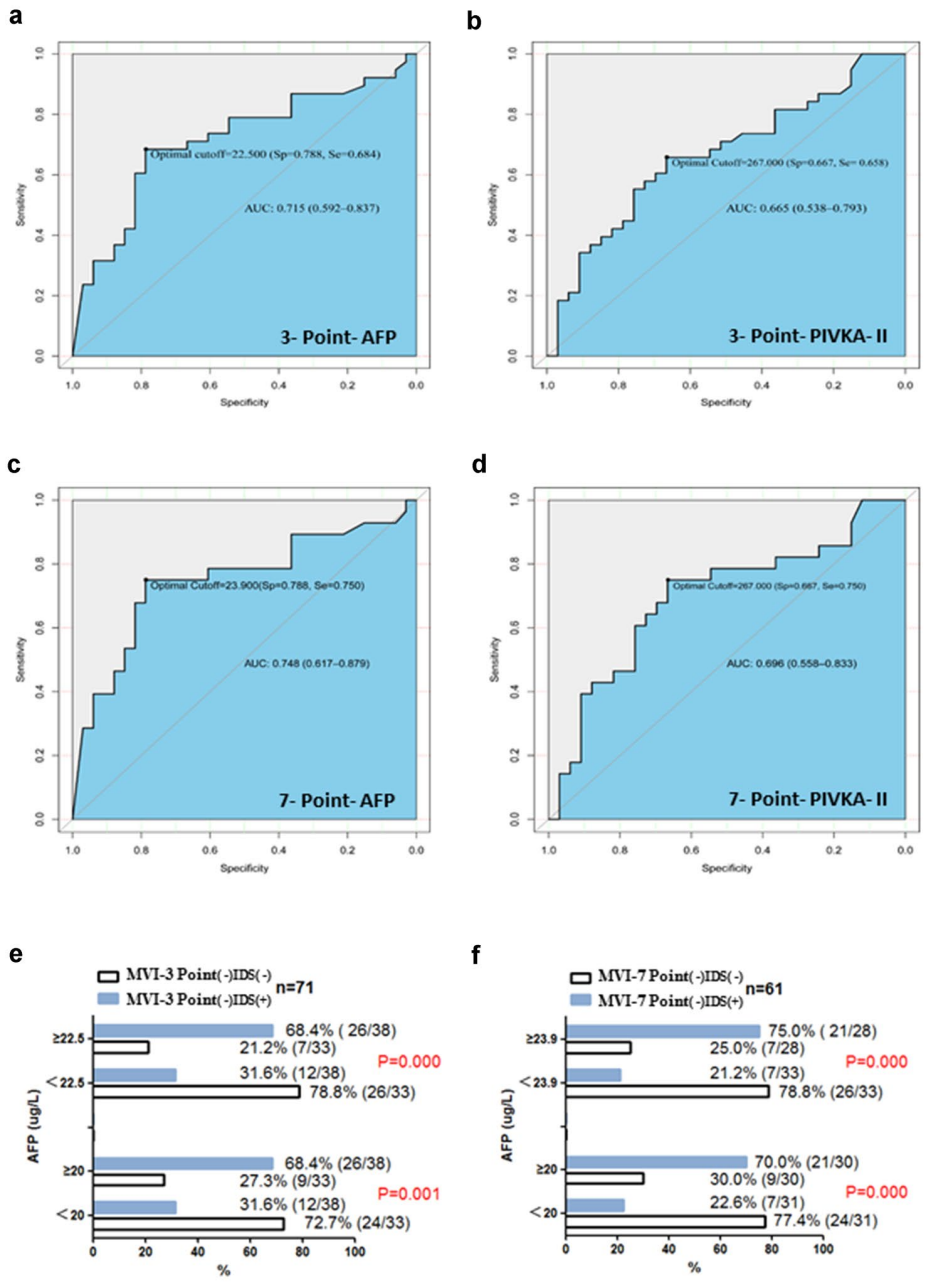
**a**, **b**, The ROC curve of AFP and PIVKA-II to predict MVI false negative status under 3-point baseline sampling protocol. **c**, **d**, The ROC curve of AFP and PIVKA-II to predict MVI false negative status under 7-point baseline sampling protocol. **e** AFP normal value and cutoff value to distinguish MVI false negative status under 3-point baseline sampling protocol. **f** AFP normal value and cutoff value to distinguish MVI false negative status under 7-point baseline sampling protocol

In order to study the sensitivity and specificity of AFP and PIVKA-II to identify MVI false negative patients, 71 patients with MVI positive and negative status in IDS but both negative in 3-Point were compared, and a total of 61 patients with MVI positive and negative status in IDS but both negative in 7-Point were compared. After calculating the best cutoff by maximizing the Youden index (Table 5), in 3-Point, when AFP was divided by a cutoff of 22.5 ng/mL, the AUC was 0.715 (0.592–0.837), the sensitivity was 0.68 (0.51–0.82), and the specificity was 0.79 (0.61–0.91). When using 267 mAU/mL as the cutoff of PIVKA-II, the AUC was 0.665 (0.538–0.793), the sensitivity was 0.66 (0.49–0.80), and the specificity was 0.67 (0.48–0.82) (Fig. 5a–b). In 7-Point, when AFP was divided by a cutoff of 23.9 ng/mL, the AUC was 0.748 (0.617–0.879), the sensitivity was 0.75 (0.55–0.89), and the specificity was 0.79 (0.61–0.91). When using 267 mAU/mL as the cutoff of PIVKA-II, the AUC was 0.696 (0.558–0.833), the sensitivity was 0.75 (0.55–0.89), and the specificity was 0.67 (0.47–0.81) (Fig. 5c–d). Therefore, AFP is superior to PIVKA-II as a biomarker to distinguish MVI false negative patients.

**Table 5:** The cutoff value of AFP and PIVKA-II to detect MVI in 3-Point and 7-Piont baseline sampling protocol

	3-Point		7-Point	
Metrics	AFP Optimal Cutoff ≥ 22.5 ng/mL	PIVKA-II Optimal Cutoff ≥ 267 mAU/mL	AFP Optimal Cutoff ≥ 23.9 ng/mL	PIVKA-II Optimal Cutoff ≥ 267 mAU/mL
Accuracy	0.73 (0.61–0.83)	0.66 (0.54–0.77)	0.77 (0.65–0.87)	0.70 (0.57–0.81)
Sensitivity	0.68 (0.51–0.82)	0.66 (0.49–0.80)	0.75 (0.55–0.89)	0.75 (0.55–0.89)
Specificity	0.79 (0.61–0.91)	0.67 (0.48–0.82)	0.79 (0.61–0.91)	0.67 (0.48–0.82)
Positive Predictive Value	0.79 (0.61–0.91)	0.69 (0.52–0.84)	0.75 (0.55–0.89)	0.66 (0.47–0.81)
Negative Predictive Value	0.68 (0.51–0.82)	0.63 (0.45–0.79)	0.79 (0.61–0.91)	0.76 (0.56–0.90)

*AFP* alpha-fetoprotein, *PIVKA-II* protein induced by vitamin K antagonist-II, *MVI* microvascular invasion, *3-Point* 3-point baseline sampling protocol, *7-Point* 7-point baseline sampling protocol

### Potential clinical utility of upper limit of AFP normal value to identify MVI false negative patients in conventional pathological sampling protocols

Because the cutoff values of AFP in 3-Point and 7-Point (22.5 and 23.9 ng/mL) were similar to the upper limit of normal value of AFP (20 ng/mL), the comparison and analysis were carried out using 22.5 ng/mL and 20 ng/mL, 23.9 ng/mL and 20 ng/mL respectively. In 3-Point sampling method, the detection rate of patients with MVI false negative status was 68.4% (26/38), and there was a significant difference (Fig. 5e, Table 6). In 7-Point sampling method (Fig. 5f, Table 7), the detection rates of patients with MVI false negative status patients were 75% (21/28) and 70% (21/30) respectively, both of which were significantly different. That is to say, in 3-Point and 7-Point, 68.4% and 70% of patients with MVI negative status and with AFP greater than 20 ng/mL are likely to be false negative.

**Table 6:** AFP normal value and cutoff value to distinguish MVI false negative status under 3-point baseline sampling protocol

Variables	Total (*n* = 71)	MVI-3P (−) IDS (−) (n = 33)	MVI-3P (−) IDS (+) (*n* = 38)	*p*	Statistic
AFP, *n* (%)				0.001	10.375
< 20 ng/mL	36 (50.70%)	24 (66.67%)	12 (33.33%)		
≥ 20 ng/mL	35 (49.30%)	9 (25.71%)	26 (74.29%)		
AFP, *n* (%)				< 0.001	12.433
< 22.5 ng/mL	39 (54.93%)	26 (66.67%)	13 (33.33%)		
≥ 22.5 ng/mL	32 (45.07%)	7 (21.88%)	25 (78.12%)		

*AFP* alpha-fetoprotein, *MVI* microvascular invasion, *3P* 3-point baseline sampling protocol, *IDS* Image-matching digital macro-slide

**Table 7:** AFP normal value and cutoff value to distinguish MVI false negative status under 7-point baseline sampling protocol

Variables	Total (*n* = 61)	MVI-7P (−) IDS (−) (*n* = 33)	MVI-7P (−) IDS (+) (*n* = 28)	*p*	Statistic
AFP, *n* (%)				< 0.001	11.962
< 20 ng/mL	31 (50.82%)	24 (77.42%)	7 (22.58%)		
≥ 20 ng/mL	30 (49.18%)	9 (30.00%)	21 (70.00%)		
AFP, *n* (%)				< 0.001	15.549
< 23.9 ng/mL	33 (54.10%)	26 (78.79%)	7 (21.21%)		
≥ 23.9 ng/mL	28 (45.90%)	7 (25.00%)	21 (75.00%)		

*AFP* alpha-fetoprotein, *MVI* microvascular invasion, *7P* 7-point baseline sampling protocol, *IDS* Image-matching digital macro-slide

## Discussion

HCC is characterized as a complex and heterogeneous disease with diverse individual outcomes. MVI is one of the most prominent features and important prognostic factors of long-term survival in HCC. The reported detection rate of MVI in HCC was relatively low in previous studies, ranging from 15.0 to 33.8% after liver resection or transplantation [12–15]. Because of the limited range of ordinary glass slices and the varied criteria in pathological sampling protocol, the conventional pathological testing approach tends to undervalue the presence of MVI in HCC. Therefore, establishing a novel and practical pathological examination approach which can increase MVI detection rate is urgently needed.

With the breakthrough of WSI techniques, high-resolution macro-pathological digital pictures can be acquired. After combining the digital pathological macro-slide data with the imaging informational of patients, the pathological information including the distribution and characteristics of MVI can be accurately mapped to the corresponding images. This novel pathological examination technique is named as IDS. The major difference of IDS and WSI is the slide formats and sizes. IDS sufficiently takes advantage of macro-slide as its bridging vehicle of pathological and imaging information.

The present study first reported the clinical utility of IDS in detecting MVI in HCC. In this study, we showed that IDS had a higher MVI detection rate than classical pathological examination approaches. In our study, the detection rate of MVI using IDS was significantly higher than conventional pathological approaches (63.7 vs. 33.0% or 22.0%), indicating that IDS had superior power in MVI detection compared with classical method.

Additionally, we provided evidence that in patients diagnosed with MVI negative status, the recurrence rate was significantly lower using IDS compared with conventional pathological method (6.1 vs. 16.4% or 16.9%), suggesting it can guide postoperative surveillance and adjuvant treatments through screening those patients with MVI false-negative status (that is, MVI was detected by IDS but undetected by conventional pathological testing) in conventional pathological methods, thus reducing long-term recurrence rates. Furthermore, IDS and conventional pathological testing could complement with each other mutually. Our findings demonstrated that patients detected as MVI positive in both IDS and conventional pathological protocols were most likely to occur disease recurrence in the near future following initial hepatic surgery. The recurrence rate of patients with MVI false-negative status was substantially higher than that of patients with actual MVI negative status.

We also selected AFP as a suitable and robust biomarker to identify MVI false-negative patients in conventional pathological protocol. Approximately 70% of HCC patients who were diagnosed as MVI negative status in conventional pathological examination may be MVI positive status in IDS testing. This encouraging result showed that IDS outperformed conventional pathological method in MVI detection for patients with abnormal AFP. For patients with normal AFP level (≤ 20 ng/mL) and MVI negative status in conventional pathological method, IDS was also a crucial way to confirm the actual MVI status.

Previously, Sheng et al. [16] proposed a standardized pathological proposal for evaluating MVI of HCC. They concluded that the MVI detection rate determined by seven-point sampling protocol (SPSP) was significantly higher than that determined by 3-point sampling method (47.1 vs. 34.5%, *p* = 0.048). Nevertheless, there was no marked difference in MVI detection rate between SPSP and 13-point sampling method (47.1 vs. 51.3%, *p* = 0.517). Therefore, we suppose that it is a futile effort to simply increase the sampling numbers beyond 7 points in conventional pathological slides. Contrarily, IDS is a useful and promising pathological technique to further increase MVI detection rates on the basis of conventional pathological testing method, thus guiding the subsequent treatment strategies of patients with MVI.

The potential application of IDS in clinical practice goes beyond MVI detection. In our on-going study, the locations of MVI can be accurately positioned using IDS, providing a reliable way to explore the distribution patterns of MVI in HCC and other tumors. Additionally, IDS can facilitate in judging the degree of tumor necrosis and observing the infiltration of inflammatory and immune cells, which can improve the efficacy evaluation of non-surgical treatments, such as transarterial chemoembolization, tyrosine kinase inhibitors and immune checkpoint inhibitors.

This study has some limitations. First, the small sample size and observational nature of this study may potentially affect the results. Second, all of the patients included in this study had a background of HBV infection. Whether IDS is applicable to patients with other etiologies of HCC needs further investigation. Third, this study is based upon our single-center data. The findings derived from this study require external validations.

## Conclusions

This study demonstrates that imaging matching digital macro-slide (IDS) first implemented by our team can help improve the detection rate of MVI in HCC and refine the prediction of HCC prognosis. It highlights the importance of establishment of a novel pathological algorithm of IDS to study HCC more comprehensively and allow for a better understanding of number and distribution of MVI under microscope. In the future, combining with artificial intelligence-driven approaches, IDS has the opportunity to be the standardized pathological examination method in all types of cancers.

## Supplementary Information

Below is the link to the electronic supplementary material.

Customized items or equipment for IDS. A, Tissue embedding box (7.7 cm × 4.3 cm × 1.3 cm). B, custom-made anti-off slide glass (7.5 cm×5.0 cm) and cover glass (6.0 cm×5.0 cm). C, special paraffin embedding mold. D, large paraffin block holder. E, special cutter head. F, Olympus Automatic Digital Pathology Scanner (VS120) (TIF 1920 KB)

The flow diagram of the preparation and production process of image-matching digital macro-slide (IDS) (TIF 4184 KB)

Another two representative HCC cases with histopathological details focused on MVI in our study (TIF 5685 KB)

## Data Availability

Data sets analyzed in the study can be provided by the corresponding author on reasonable request.
