# Supplementary figures and images for: Image-matching digital macro-slide—a novel pathological examination method for microvascular invasion detection in hepatocellular carcinoma
Source: Hepatol Int. 2022 Mar 16;16(2):381–95. doi: 10.1007/s12072-022-10307-w (PMC9013327; doi:10.1007/s12072-022-10307-w)

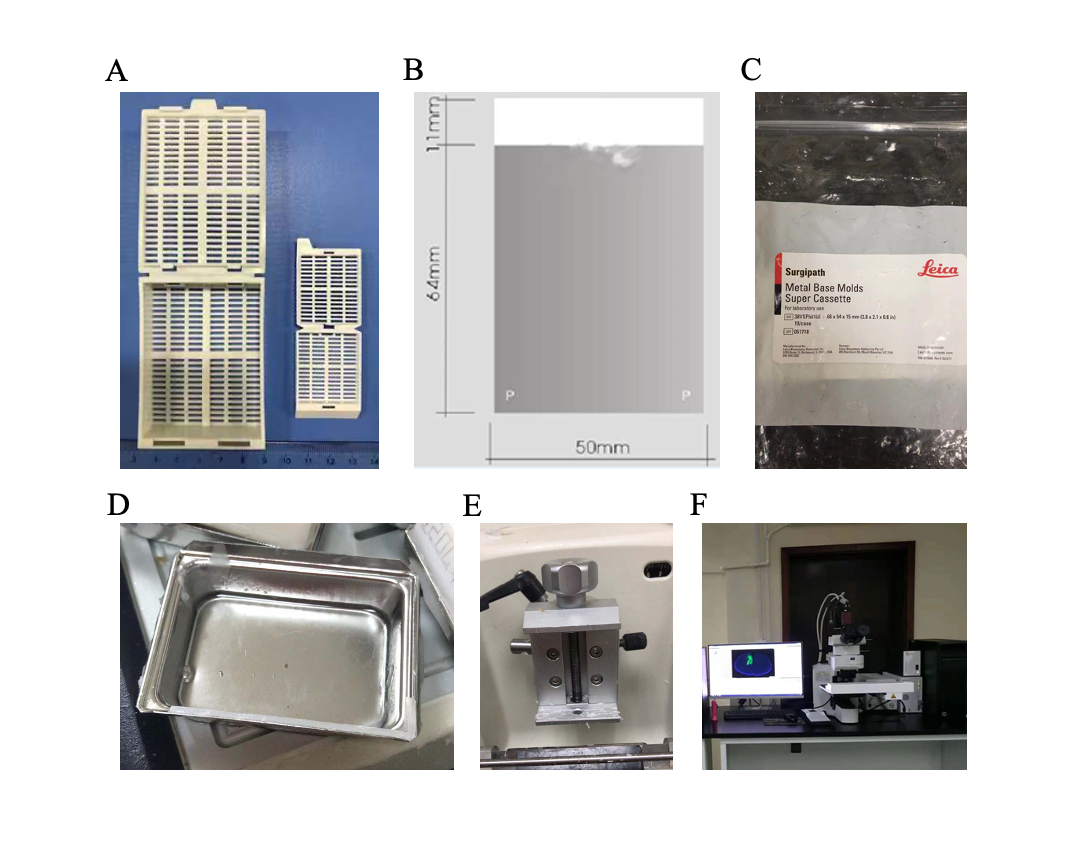

Supplement: Supplementary file 1 — Customized items or equipment for IDS. A, Tissue embedding box (7.7 cm × 4.3 cm × 1.3 cm). B, custom-made anti-off slide glass (7.5 cm×5.0 cm) and cover glass (6.0 cm×5.0 cm). C, special paraffin embedding mold. D, large paraffin block holder. E, special cutter head. F, Olympus Automatic Digital Pathology Scanner (VS120) (TIF 1920 KB) [file 12072_2022_10307_MOESM1_ESM.tif]

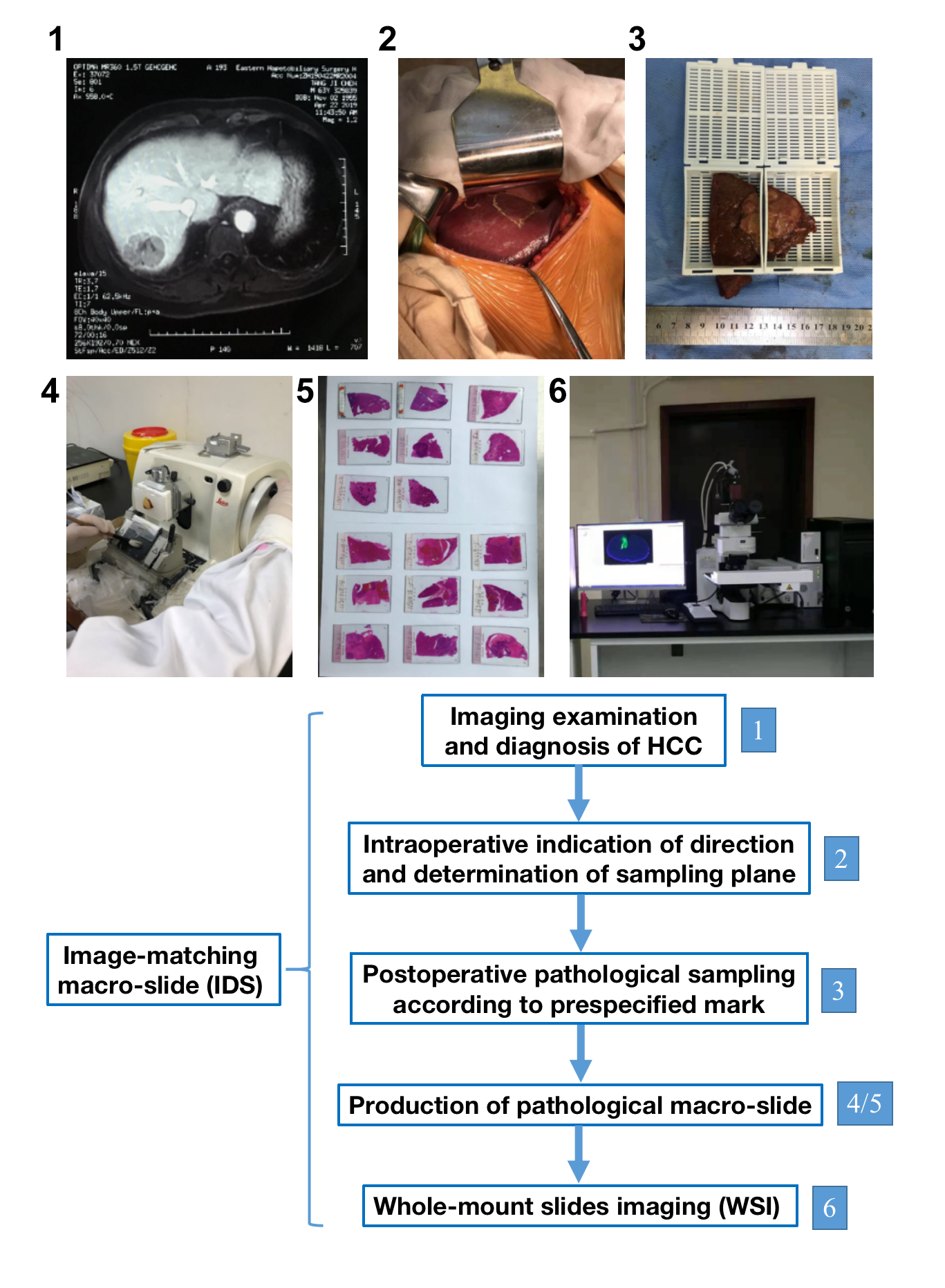

Supplement: Supplementary file 2 — The flow diagram of the preparation and production process of image-matching digital macro-slide (IDS) (TIF 4184 KB) [file 12072_2022_10307_MOESM2_ESM.tif]

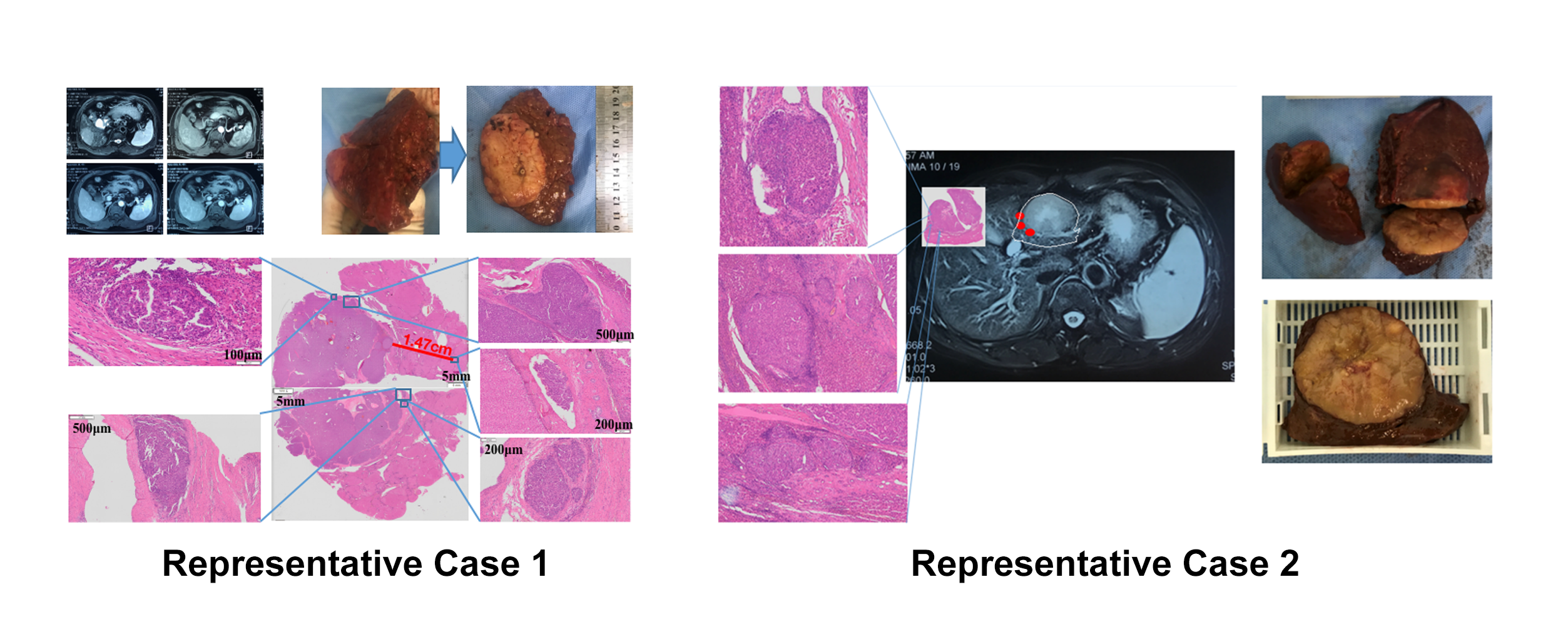

Supplement: Supplementary file 3 — Another two representative HCC cases with histopathological details focused on MVI in our study (TIF 5685 KB) [file 12072_2022_10307_MOESM3_ESM.tif]
